# Polymorphisms in Glutathione S-Transferase (*GST*) Genes Modify the Effect of Exposure to Maternal Smoking Metabolites in Pregnancy and Offspring DNA Methylation

**DOI:** 10.3390/genes14081644

**Published:** 2023-08-18

**Authors:** Parnian Kheirkhah Rahimabad, A. Daniel Jones, Hongmei Zhang, Su Chen, Yu Jiang, Susan Ewart, John W. Holloway, Hasan Arshad, Shakiba Eslamimehr, Robert Bruce, Wilfried Karmaus

**Affiliations:** 1 Division of Epidemiology, Biostatistics, and Environmental Health Sciences, School of Public Health, University of Memphis, Memphis, TN 38111, USA; parnian.k@memphis.edu (P.K.R.); hzhang6@memphis.edu (H.Z.); yjiang4@memphis.edu (Y.J.); sslmmehr@memphis.edu (S.E.); 2 Department of Biochemistry & Molecular Biology, Michigan State University, East Lansing, MI 48824, USA; jonesar4@msu.edu; 3 Department of Biostatistics, College of Public Health, University of Nebraska Medical Center, Omaha, NE 68198, USA; suchen@unmc.edu; 4 Department of Large Animal Clinical Sciences, Michigan State University, East Lansing, MI 48824, USA; 5 Human Development and Health, Faculty of Medicine, University of Southampton, Southampton SO17 1BJ, UK; j.w.holloway@soton.ac.uk; 6 Clinical and Experimental Sciences, Faculty of Medicine, University of Southampton, Southampton SO17 1BJ, UK; s.h.arshad@soton.ac.uk; 7 The David Hide Asthma and Allergy Research Centre, Isle of Wight, Newport PO30 5TG, UK; 8 NIHR Southampton Biomedical Research Centre, University Hospital Southampton, Hampshire, Southampton SO16 6YD, UK; 9 Department of Anesthesiology, College of Medicine, University of Tennessee Health Science Center, Memphis, TN 38163, USA; rbruce5@uthsc.edu

**Keywords:** smoking, pregnancy, DNA methylation, genetic polymorphism, glutathione S-transferase

## Abstract

Maternal smoking in pregnancy (MSP) affects the offspring’s DNA methylation (DNAm). There is a lack of knowledge regarding individual differences in susceptibility to exposure to MSP. Glutathione S-transferase (*GST*) genes are involved in protection against harmful oxidants such as those found in cigarette smoke. This study aimed to test whether polymorphisms in *GST* genes influence the effect of MSP on offspring DNAm. Using data from the Isle of Wight birth cohort, we assessed the association of MSP and offspring DNAm in 493 mother-child dyads (251 male, 242 female) with the effect-modifying role of *GST* gene polymorphism (at rs506008, rs574344, rs12736389, rs3768490, rs1537234, and rs1695). MSP was assessed by levels of nicotine and its downstream metabolites (cotinine, norcotinine, and hydroxycotinine) in maternal sera. In males, associations of hydroxycotinine with DNAm at cg18473733, cg25949550, cg11647108, and cg01952185 and norcotinine with DNAm at cg09935388 were modified by *GST* gene polymorphisms (*p*-values < 0.05). In females, associations of hydroxycotinine with DNAm at cg12160087 and norcotinine with DNAm at cg18473733 were modified by *GST* gene polymorphisms (*p*-values < 0.05). Our study emphasizes the role of genetic polymorphism in *GST* genes in DNAm’s susceptibility to MSP.

## 1. Introduction

Despite being associated with a multitude of adverse health outcomes both for the mother and the offspring, maternal cigarette smoking in pregnancy (MSP) is prevalent around the world [1]. According to a global meta-analysis, the prevalence of MSP exceeded 10% in 29 of 174 countries and exceeded 20% in 12 other countries [1]. In Western countries, the estimated prevalence of MSP is 8%, showing significant variation between countries: 16% in France, 12% in the UK, 9% in Germany, and 7% in the US, respectively [2].

Epidemiological studies have shown that offspring exposed to in-utero cigarette smoking are at increased risk of adverse development of the respiratory system [3]. MSP is a leading preventable cause of suboptimal in-utero development of the lungs, leading to a future risk of decreased lung function parameters, wheezing, asthma, and respiratory infections [3]. Awareness of the newborn’s prenatal exposure to cigarette smoking is critical to identifying those at risk of developing adverse health outcomes [4].

Cigarette smoke is composed of a wide variety of toxic chemicals such as ammonia, benzene, acrolein, carcinogenic nitrosamines, quinones, polycyclic aromatic hydrocarbons [5], and nicotine [6]. Nicotine is a major addictive ingredient in tobacco plant leaves that impedes smokers’ efforts to quit [7]. After inhalation of cigarette smoke, nicotine is absorbed from the lungs’ alveoli and reaches the bloodstream. It is then metabolized by the liver enzyme CYP2A6 to cotinine, a downstream metabolite [7]. Nicotine and its downstream metabolites such as cotinine, norcotinine, and hydroxycotinine cross the placenta and reach the fetal bloodstream [8]. Nicotine and its metabolites have been used as biomarkers of smoking exposure. Since nicotine has a short half-life (1–2 h) and is quickly removed from the bloodstream, its downstream metabolites such as cotinine, norcotinine, and hydroxycotinine might provide more reliable biomarkers (half-life: 13–19 h) [8].

Although epidemiological studies have shown adverse effects of MSP on offspring health, the underlying mechanisms remain unclear. One potential mechanism is through changes in DNA methylation (DNAm) [9]. This involves transferring methyl groups to cytosine. Changes in DNAm can be detected on cytosines preceding a guanine nucleotide, called ‘CpG sites’ [10]. These C-G rich segments of the DNA influence gene expression through their levels of methylation [11]. In addition, DNAm has been shown to have an impact on gene expression by impeding the binding of transcription factors to the DNA and on alternative splicing by marking exons through hypermethylation [12]. Methylation of DNA can also occur in the bodies of genes such as the hydrocarbon receptor repressor (*AHRR*) gene involved in the metabolism of toxic substances, including those in cigarette smoke [13], although the functional consequences of this methylation are still unknown. Through these mechanisms, DNAm leads to variations in metabolic processes and cellular behavior [14].

It is assumed that during the formation of the zygote, DNAm marks are removed [15]. Subsequently, DNAm is reestablished during embryonic development [16]. DNAm is critical for the differentiation of cells into distinct lineages from a single zygote [16]. Additionally, DNAm has been linked to the development of a range of diseases and other health conditions [16]. The establishment of DNAm is highly influenced by environmental factors to which the growing embryo is exposed [17].

Prenatal smoking exposure has been shown to affect offspring DNAm in several studies [2]. In a meta-analysis by Joubert et al., over 6000 differentially methylated CpG sites were identified to be associated with MSP [18]. Given the effect of prenatal smoking exposure on the offspring’s DNAm and the potential association of differential DNAm with health conditions, Joubert et al. suggested that influencing DNAm could be a potential mechanism by which prenatal smoking exposure affects the risk of future adverse health outcomes [18].

However, one limitation of prior studies is the lack of knowledge on the impact of individual genetic differences on the association of MPS with DNAm. For instance, Glutathione S-transferase (*GST*) represents a gene family that encodes several proteins involved in the detoxification of environmental pollutants and protection against reactive oxygen species (ROS) [19]. Studies have shown that some *GST* genes, such as *GSTA1*, *GSTP1*, and *GSTM1*, are highly expressed in the fetal liver and are responsible for mitigating the effects of ROS produced as a result of exposure to toxic substances [20,21]. This study aimed to test whether the effects of MSP on offspring DNAm are influenced by their *GST* genetic polymorphism. We used data from the Isle of Wight birth cohort (IoWBC) to examine whether *GST* gene polymorphisms have a modifying role in the association between MSP and DNAm.

## 2. Materials and Methods

### 2.1. Study Population

The IoWBC is a British birth cohort established in 1989 to investigate asthma and allergic disorders on the Isle of Wight, UK [22]. The birth cohort has been approved by both the local research ethics committee, specifically the NRES Committee of South Central—Hampshire B, UK, and the University of Memphis IRB (Institutional Review Board) in Memphis, U.S. (STUDY number: 2423). All individuals participating in recruitment and follow-ups gave parental or personal written consent.

A total of 1536 children born between 1 January 1989 and 28 February 1990, were identified on the Isle of Wight. After excluding those who declined to participate, perinatal deaths, and adoptions, 1456 remained in the study (F1 generation). Parents of the cohort members (F1 generation) were included as the F0 generation. Mother-child dyads (F0 mother-F1 child) were included in this study.

### 2.2. Assessment of Exposure: MSP

Information on MSP was obtained during pregnancy using questionnaires. In addition, nicotine and its metabolites were measured from maternal blood serum drawn at delivery.

#### Assessment of Nicotine and Its Downstream Metabolites in Maternal Sera

Nicotine and its downstream metabolites, cotinine, norcotinine, and hydroxycotinine, were assessed in maternal sera as markers of cigarette smoking during pregnancy. Processing and analysis of maternal serum specimens were carried out in random order. Maternal sera at delivery (20 µL aliquots) were obtained using a modified version of the Matyash protocol [23] after addition of 25 pmol cotinine-*d*_3_ as an internal standard and metabolites partitioned into water- and organic-soluble fractions. After separation of the fractions, a SpeedVac vacuum centrifuge was used without applying heat to evaporate solvent from the lower (polar) fraction. The collected residues were dissolved in 200 µL of acetonitrile/water and subsequently transferred to an auto-sampler vial with a 200-microliter insert.

Polar fraction metabolites were profiled using LC/high resolution MS on a QExactive mass spectrometer (Thermo Electron North America LLC, Madison, WI, USA) with a Thermo Vanquish Flex binary pump. An auto-sampler equipped with an Acquity BEH Amide column (measures: 10 cm × 1.0 mm, 1.7 µm, Waters, Milford, MA, USA) for hydrophilic interaction liquid chromatography (HILIC) separation was used. Analyses were performed in positive-ion mode with full scan/all-ion fragmentation. Chromatographic separations were carried out using a gradient based on solvent A (100 mM ammonium acetate + 0.4% ammonium hydroxide, pH 9.0 before mixing) in acetonitrile/water (1:1 *v*/*v*) and Solvent B (containing 100 mM ammonium acetate + 0.04% ammonium hydroxide, adjusted to pH 9.0 before mixing) in acetonitrile/water (9:1 *v*/*v*). Gradient was 0.0–1.0 min (99% B); 7.0–10.0 min (50% B); 10.01 min (99% B); hold until 15 min. The process of aligning, detecting, and normalizing serum constituents was carried out using Progenesis QI v2.4 software provided by Waters, Nonlinear Dynamics, located in Newcastle upon Tyne, NE1 2JE, UK. For metabolite annotation, a search of the extracted spectra was carried out using Compound Discoverer software (Thermo) and the mzCloud database (Thermo). Fragment ions presence characteristics in the high collision-energy mass spectra were manually verified. From the Progenesis software, peak areas were exported. Furthermore, the dataset was filtered to remove signals with a high number of blanks and those with RMD (relative mass defect) > 1200 ppm [24,25], which are generally inorganic salts. Peaks after export were normalized based on areas of internal standard cotinine-*d*_3_. They were scaled by multiplying by 1 × 10^4^. Due to the similar properties of nicotine and its downstream metabolites, their levels were all normalized by multiplication by 0.125 in order to convert the signals to nM concentrations in the sera.

Nicotine and some of its metabolites exhibited a relatively high percentage of zero values, exceeding 30%. These zeros could be attributed to either technical factors, such as values falling below the limit of detection or accidental errors in detecting peak or thresholding, or biological factors, such as zero or near-zero abundance in non-smokers. To handle the substantial number of values below the detection limit while minimizing the impact on relatively rare exposure markers, we could have employed a QRILC approach (quantile regression imputation of left-censored data) [26] for the imputation of missing values. This approach, however, would introduce a challenge. The metabolite levels exhibit strong right-skewness due to the presence of severe outliers. If we treat measurements of metabolites as continuous data, it would be necessary to apply a log transformation before conducting any statistical analyses that depend on normality assumptions. However, if a significant proportion of zeros (>30%) were imputed with small random values, the log transformation can amplify the influence of these artificially imputed values, potentially biasing the estimation of parameters in subsequent analyses. Rather than imputing the excessive zeros (>30%), we decided to rank nicotine and its metabolites based on signal abundances using the PROC RANK in SAS, allowing for up to five ranks (0/1/2/3/4). This conservative approach also helps minimize the impact of outliers [27]. To control for the variation among batches of nicotine and its metabolites, analyses were adjusted by a batch variable.

### 2.3. Assessment of the Outcome: DNAm of the F1 Offspring

DNA from the F1 generation was extracted from dried blood spots obtained from heel prick tests collected on Guthrie cards after birth following the procedure by Beyan et al. [28]. Briefly, QIAamp DNA Investigator kits (Qiagen Inc., Germantown, MD, USA) were utilized for DNA extraction from three 6mm samples that were punched from Guthrie cards following the manufacturing company’s protocol. The measurement of DNA concentration was performed using a Qubit spectrophotometer. Samples with a concentration equal to or higher than 1.14 ng/uL were chosen for further processing. Infinium Methylation EPIC BeadChip arrays (Illumina Inc., San Diego, CA, USA) were used for DNAm measurements, which provided about 850,000 DNAm sites. There were eight total batches from the F1 generation’s DNAm data. DNAm intensities underwent quality control and pre-quantile normalization following the CPACOR pipeline [29]. ComBat [30] was used to remove the batch effect. In addition, we excluded CpG sites containing probe-SNPs within ten base pairs and those CpGs that had a MAF (minor allele frequency) less than 0.007. Finally, 551,710 CpGs remained for further analysis. DNAm levels in β values were assigned to each CpG locus based on the BeadStudio software methylation module. β values (β = methylated/(methylated + unmethylated + c)) signify the proportions of methylated on the total methylated and unmethylated CpG loci. Herein, c is a constant to avoid division by zero [28]. M values were calculated as the logit-transformed β-values of DNAm (M = log2 (β/1 − β)). In regression analyses, M values were used to alleviate severe heteroscedasticity in β values [31].

Because of the different compositions of cell-type populations, analyses were adjusted for proportions of cell types to remove their confounding effect on DNAm assessment [32]. For estimating cell-type proportions of blood, the R-package “minfi” was used [33]. Eosinophils’ proportions were further estimated indirectly using a DNAm profile based on the Houseman approach [34] incorporated into the “minfi” R package. We adjusted for estimations of cell-type proportions including CD4+ T, B-cells, monocytes, natural killer cells, neutrophils, and eosinophils in statistical models using DNAm data.

This investigation focuses on DNAm CpG sites known to be associated with maternal smoking. Hence, CpGs were extracted from the meta-analysis by Joubert et al. [18]. Based on the meta-analysis of data from 13 cohorts using the Illumina 450 k methylation array, Joubert et al. identified 6073 CpG sites that had a significant association with MSP, of which 568 survived Bonferroni adjustments [18]. Among the 551,710 CpGs in the IoWBC, 460 of the 568 CpGs were available and included in our analyses.

### 2.4. Assessment of Covariates

Due to sex-specific differences in DNAm [35,36], analyses were conducted with a sex-stratified approach. Covariates used in all models include maternal age at delivery, maternal body mass index (BMI), and the child’s socioeconomic status (SES) during childhood/adolescence. The weight and height of the mothers were assessed during pregnancy and used to calculate BMI (BMI = weight (kg)/height^2^ (m)). Hospital records were used to obtain mothers’ ages at delivery and the gender of their offspring. SES was derived from three variables: British socioeconomic classes determined by the occupation of the parents (1–6), the total income of the family, and the number of children residing in the bedroom of the index child. SNPs of the *GST* gene were evaluated as potential effect modifiers of the association between nicotine and its metabolites and offspring DNAm.

### 2.5. Assessment of Effect Modifiers (GST Gene Polymorphisms)

DNA was extracted from the whole blood or saliva of IoWBC participants for genotyping (N = 1211), as previously described [37]. Single nucleotide Polymorphisms (SNPs) in *GST* genes were genotyped using Illumina GoldenGate assays [38]. *GST* SNPs with available data were rs929166, rs10735234, rs11807, rs12024479, rs12736389, rs1537234, rs1537236, rs1695, rs366631, rs3768490, rs506008, rs560018, rs574344, rs638820, and rs7483.

To decrease the number of multiple tests, particularly for correlated SNPs, the number of SNPs was reduced to haplotype blocks based on linkage disequilibrium (LD) [39]. To identify haplotype blocks, Haploview [40] was used. The *GST* SNPs were statistically grouped into six blocks of LD (Table 1). SNPs used in the analysis are underlined. The SNP rs1695 did not belong to any block but represented the *GSTP1* gene and was analyzed accordingly.

### 2.6. Statistical Analysis

All analyses were carried out using the F0 (mothers) and F1 (offspring) generations of the IoWBC using SAS (version 9.0) and Haploview [40]. To show the differences in the methylation proportion for different levels of the nicotine metabolites, we compared the DNAm of the lowest with the highest rank of the metabolites.

The association of nicotine and its metabolites with offspring DNAm was assessed using linear regression models with nicotine and its metabolites in maternal sera as the predictors, the offspring *GST* SNP as a predictor and potential effect modifier, and the DNAm of the offspring as the outcome. The linear regression models were adjusted with the following covariates: maternal age, BMI, SES, and a variable presenting batch groups of nicotine metabolites.

To assess the goodness of fit of the regression models, the explained variance (R^2^) was calculated for three models. Model 1 included nicotine and its metabolites and confounders. Model 2 additionally addressed the *GST* SNPs. Model 3 included nicotine metabolites, *GST* SNPs, and the interaction between nicotine metabolites and *GST* SNPs. Model 1 explains how much variability in the offspring’s DNAm is related to exposure to nicotine and its metabolites. Comparing models 1 and 2 shows whether *GST* polymorphisms add to the explanatory power of the model. Comparing models 3 and 2 shows the role of interaction terms in explaining a part of the DNAm variability.

The False Discovery Rate (FDR) method [25] was utilized to account for multiple testing during the assessment of the association between nicotine metabolites and offspring DNAm [32]. A significance level of *p* < 0.05 was employed to determine statistical significance.

## 3. Results

After excluding those with missing information on biomarkers of MSP or offspring DNAm, 493 mother-child dyads from F0–F1 generations were included in the study. There was no statistical difference in characteristics between the analyzed sample and the total cohort for males and females (Table 2).

The SNP rs506008 has three genotype groups: AA, AC, and CC (Table 3). Since AA was a rare occurrence (3 in 233 males and 4 in 227 females, respectively), it will cause a violation of the 5% rule for the chi-square test or a reduced sample size for regression analysis. Due to the rarity of individuals with the AA genotype, we combined AA with AC.

Similarly, two other *GST* SNPs (rs574344 and rs12736389) had sparse data for one of their variants. For rs574344, the AA genotype was combined with AT and compared to TT. For rs12736389, the CC genotype was added to CG and compared to GG.

To check if combining the rare genotype affects the results of the regression analysis, we further assessed the three regression models with and without the combination of the rare variant and excluding the rare variant. For example, in males, the rs12736389 polymorphism (genotypes: CC, CG, and GG) is an effect modifier of the association between maternal serum norcotinine and offspring DNAm level at cg09935388. Three regression models were run with (a) CC separated from CG, (b) CC combined with CG, and (c) CC removed from the dataset. The effect estimates and *p* values of the CG group compared to the reference group (GG) were (a) β = 0.334, *p* = 0.098; (b) β = 0.335, *p* = 0.096; and (c) β = 0.334, *p* = 0.098. The effect estimates and *p* values of the interaction term were (a) β = −0.174, *p* = 0.04; (b) β = −0.176, *p* = 0.04; and (c) β = −0.174, *p* = 0.04. Since combining the CC genotype with the CG genotype did not change the statistics, we took the approach explained above.

*GST* gene polymorphisms did not affect the concentrations of nicotine metabolites in maternal serum for any of the blocks (Supplementary Table S1), nor was there any interaction of haplotype blocks with MSP for the concentration of nicotine metabolites in maternal serum. These findings simplified the analyses on whether (b) *GST* gene polymorphism of the offspring modifies the association of exposure to nicotine and its metabolites with offspring DNAm since there is no need to account for the impact of *GST* SNPs on nicotine metabolites in the analysis.

Next, a potential effect modification of *GST* gene polymorphism on the association between nicotine metabolites in maternal serum (exposure) and offspring DNAm (outcome) was assessed, adjusting for effect confounders and controlling for multiple testing (FDR). From the meta-analysis by Joubert et al. [19], 460 CpGs were available in our DNAm dataset (850 k), which were used for further analysis.

Table 4 shows the CpG sites whose methylation levels were associated with MSP and with effect modification of *GST* gene polymorphisms (after FDR adjustment) for male and female offspring, respectively (only statistically significant associations are shown).

Nicotine levels were not associated with differential DNAm at any of the 460 CpG sites. However, cotinine, norcotinine, and hydroxycotinine levels were associated with different levels of offspring DNAm; however, only norcotinine and hydroxycotinine showed significant interactions with *GST* SNPs on DNAm. For male offspring, the associations of hydroxycotinine with DNAm at cg18473733 (rs574344; *GSTM2*), cg25949550 (rs574344; *GSTM2*), cg11647108 (rs1695; *GSTP1*), cg01952185 (rs1695; *GSTP1*), and maternal norcotinine with DNAm at cg09935388 (rs12736389; *GSTM5*) were significantly modified by *GST* SNPs. For female offspring, the associations of hydroxycotinine with DNAm at cg12160087 (rs506008; *GSTM4*, rs1537234; *GSTM3*) and norcotinine with DNAm at cg18473733 (rs506008; *GSTM4*) were significantly modified by *GST* SNPs.

**Figure 1:**
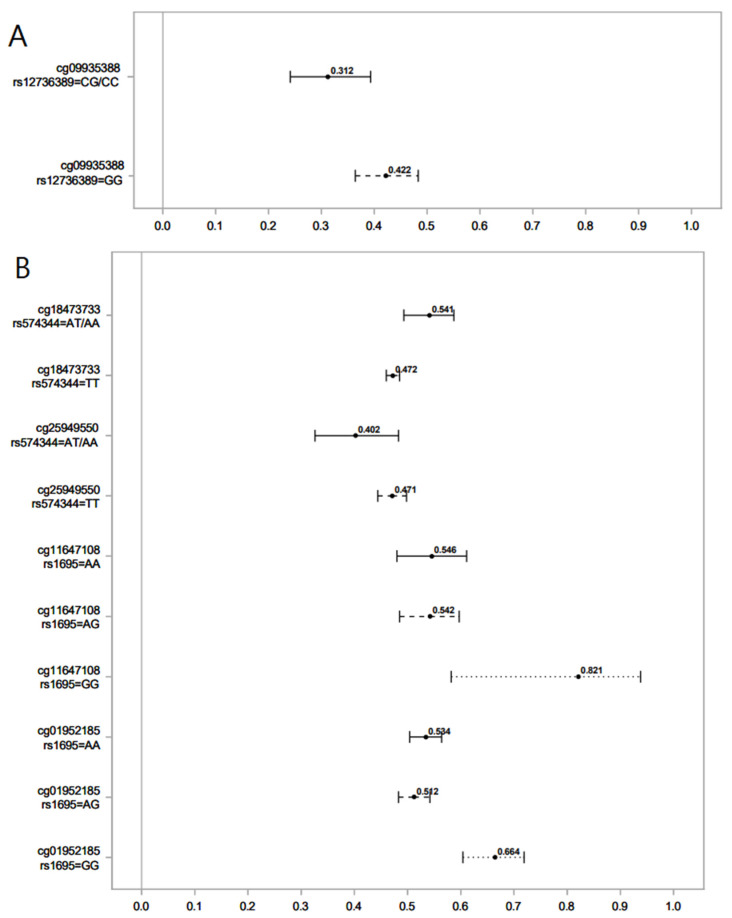
Association of serum levels of norcotinine (**A**) and hydroxycotinine (**B**) in maternal sera and male offspring DNAm for different SNPs of the *GST* gene. Lines show the proportion of methylation and their 95% CI for the highest vs. lowest rank of hydroxycotinine. Statistically important differential DNAm levels were identified using M-values; however, to present them as proportions, we used β values in the graphs. Due to the rarity of the AA genotype for rs574344, for analysis, individuals with this genotype were combined with those with the AT genotype. Due to the rarity of the CC genotype for rs12736389, individuals with this genotype were combined with those with the CG genotype.

**Figure 2:**
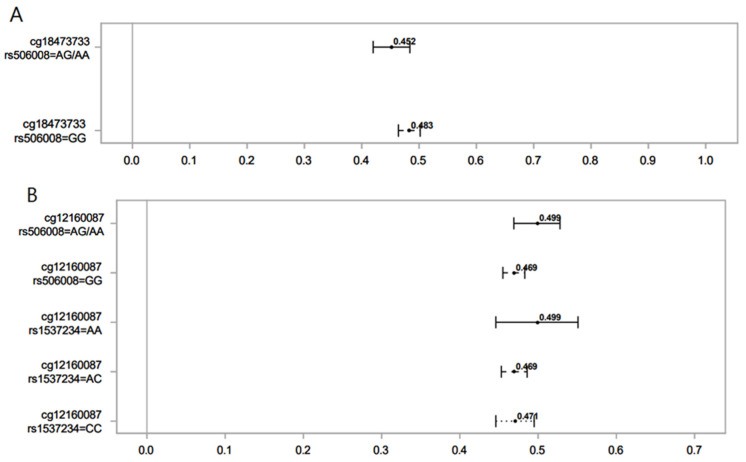
Association of norcotinine (**A**) and hydroxycotinine (**B**) in maternal sera and female offspring DNAm for different SNPs of the *GST* gene. Lines show the proportion of methylation and their 95% CI for the highest vs. lowest rank of hydroxycotinine. Statistically important differential DNAm levels were identified using M-values; however, to present them as proportions, we used β values in the graphs. Due to the rarity of the AA genotype for rs506008, individuals with the AA genotype were combined with the AG genotype.

The following graphs (Figure 1 and Figure 2) compare the difference in DNAm (β-values or proportion of methylation) levels in the highest versus lowest ranks of hydroxycotinine and norcotinine for different SNPs and genotypes. The mean difference in DNAm levels and their 95% confidence interval comparing the highest rank (4) vs. the lowest rank (0) for the interaction of the nicotine metabolite groups with *GST* genes were calculated.

The explained variance (R square) of the DNAm was calculated for regression models with and without *GST* SNP and the interaction term between nicotine metabolite and *GST* SNP (Table 5).

In regression models with norcotinine or hydroxycotinine as predictors and DANm as outcome, the addition of *GST* polymorphism increased R^2^. The addition of the interaction term between nicotine metabolite and *GST* SNP further increased R^2^ for both male and female offspring. For example, in males, the addition of the rs1695 SNP and the interaction term with hydroxycotinine increased the R^2^ by 39.6% (from 0.182 to 0.254) for DNAm at cg11647108. In females, the addition of the rs1537234 SNP and the interaction term with hydroxycotinine increased R^2^ by 27.5% (from 0.167 to 0.213) for DNAm at cg12160087.

## 4. Discussion

Since the concentrations of the four smoking metabolites in maternal sera were not affected by the *GST* genotypes of the offspring, associations between nicotine metabolites, *GST* genotypes, and their interactions on DNAm do not need adjustment for varying levels of nicotine metabolites due to *GST* enzyme activity. This is not particularly surprising in that *GST*s have not been documented to play important direct roles in nicotine metabolism but are likely involved in the metabolism of other tobacco smoke chemicals.

The results suggest that associations of nicotine metabolites in maternal sera with offspring DNAm are partially influenced by *GST* gene polymorphism and their interaction with tobacco constituent metabolites. Effect modification was observed for several members of the *GST* gene family. Hence, the role of the enzymes encoded by the *GST* gene family in the protection of the developing fetus from oxidative stress suggests individual susceptibility to MSP based on genetic polymorphism.

We found that the interaction term improved the explained variance (R^2^) for all five CpGs in males and two CpGs in females, respectively. This implies that for some CpGs, the addition of interaction terms from *GST* gene polymorphisms with nicotine metabolites improves the explanation.

We observed that the effect of modifications of *GST* SNPs on the association between nicotine metabolites and offspring DNAm is sex-specific. This might be explained by differences in the response of the placenta to oxidative stress based on the offspring’s sex [41]. Research has indicated that when exposed to unfavorable maternal conditions characterized by oxidative stress, the male placenta tends to exhibit a more prominent response compared to the female placenta [42]. In rodents, high oxidative stress conditions caused sexually dimorphic changes in placental morphology, gene expression, and enzymes involved in DNAm [41]. Furthermore, glutathione metabolism is reported to be different in male and female offspring [43]. O’Shaughnessy et al. have shown that MSP affects the transcription of some *GST* genes in the fetal liver (*GSTA1*, *GSTP1*, and *GSTM1*) in a sex-dependent manner [44].

We found that in male offspring, the AT genotype of rs574344 (*GSTM2* gene) attenuated the decrease in DNAm at cg18473733 by hydroxycotinine, but it amplified the decrease in DNAm at cg25949550 by hydroxycotinine. Genetic polymorphisms of the *GSTM2* gene at different loci, including rs574344, have been studied in association with offspring lung function after exposure to MSP [45]. No study, however, has investigated the modifying role of *GST* SNPs on the association between MSP and offspring DNAm. The CpGs cg18473733 and cg25949550 belong to the genes *KLF2* and *CNTNAP2*, respectively. The Krüppel-like factor 2 (*KLF2*) gene codes for a transcription factor that plays a crucial role during the development of embryonic vasculature [46]. The Contactin-Associated Protein 2 (*CNTNAP2*) gene is involved in nervous system development and has been implicated in disorders such as autism and intellectual disability in association with exposure to MSP [47]. The potential effect of *GSTM2* polymorphism on health outcomes in offspring exposed to MSP by altering specific DNAm remains to be addressed by future studies.

In males, the CG genotype of rs12736389 (*GSTM5*) amplified the decrease in DNAm at cg09935388 by norcotinine. This CpG belongs to the growth factor independent 1 transcriptional repressor (*GFI1*) gene and plays a role in developmental processes, including hematopoiesis [48]. DNAm at cg09935388 has been reported to mediate the effects of exposure to MSP on offspring birth weight [49]. The genetic polymorphism of *GST5* has been associated with lung function measures (FEV1 and FVC) in interaction with MSP exposure [38].

The AA and AG genotypes of rs1695 (*GSTP1*) attenuated the increase in DNAm at cg11647108 (*ANXA11*) and cg01952185 (*TIFAB*) by hydroxycotinine. The gene *ANXA11* codes for Annexin A11, a member of Annexins, i.e., phospholipid-binding proteins regulated by calcium with significant involvement in various cellular processes such as the cell life cycle, exocytosis, and apoptosis [49]. TRAF-interacting protein with a forkhead-associated domain B (*TIFAB*) is another protein-coding gene implicated in several cellular signaling pathways involved in hematopoiesis [50]. Structural polymorphisms in the *GSTP1* gene have been reported to affect the association between MSP and offspring health outcomes by altering the encoded enzyme activity [51]. The genotypes AA and AG of rs1695 (*GSTP1*) have been shown to increase the risk of early life wheezing in children exposed to MSP [51].

In female offspring, hydroxycotinine decreased DNAm at cg12160087. However, offspring with an AG genotype of rs506008 (*GSTM4*) had higher DNAm levels compared to GG. Those with AA and AC SNPs of rs1537234 (*GSTM3*) had higher and lower DNAm levels compared to CC, respectively. DNAm levels at cg12160087 in the latter group (AC genotype of rs1537234) were not significantly different from the CC genotype of rs1537234. The CpG site cg12160087 is associated with the Coiled-coil domain containing 64 (*CCDC64*), a protein-coding gene involved in cellular transport and nervous system development [52]. DNAm at cg12160087 has been positively associated with offspring birth weight [53]. Previous research shows that genetic polymorphisms of the *GSTM3* gene affect lung function (FEV1 and FVC) by interacting with MSP [38].

Norcotinine was associated with decreased DNAm levels at cg18473733 (*KLF2*). The AG genotype of rs506008 (*GSTM4*) further lowered the levels of DNAm compared to the GG SNP. This CpG site has also been observed to be significantly associated with MSP in male offspring. *GSTM4* has not been well studied in association with health outcomes [45]. Variations in *GSM4* have been linked to lung function measures [45] and lung cancer [54].

In this study, we focused on the CpGs whose DNAm was associated with nicotine metabolites, with a significant effect-modifying role for *GST* SNPs. However, there are CpGs associated with nicotine metabolites for which we did not find a significant effect-modifying role for *GST* polymorphism. This may imply that maternal nicotine metabolites may serve as surrogates for tobacco smoke chemicals that affect offspring DNAm through different biological mechanisms in addition to increasing oxidative stress. One of these CpGs is cg05575921 (in the body of the *AHRR* gene), whose lower methylation has been reported consistently as a marker of exposure to MSP [55,56]. Although significantly associated with nicotine metabolites, DNAm at cg05575921 was not influenced by *GST* SNPs in our study. This might further emphasize its usefulness as a smoking exposure biomarker since it is not influenced by individual differences in *GST* genotype.

To the best of our knowledge, our study is the first to address the role of *GST* gene polymorphisms in modifying the associations of nicotine metabolites with offspring DNAm. Using a prospective design and a population-based cohort are among the strengths of our study. Additionally, we used a more objective assessment for MSP, i.e., nicotine and its downstream metabolites in maternal serum.

There are some limitations in our study worth mentioning. First, from the 586 CpGs extracted from the Joubert et al. meta-analysis, we had information on 460 CpGs. Hence, some of the CpGs not included in our quality control might have been associated with nicotine metabolites through the effect-modifying role of *GST* SNPs. Second, our data on the *GST* gene was limited to different *GSTM* genes and one *GSTP* gene. Although most *GST* genes associated with MSP belong to these two groups, there are previous studies that suggest a potential role for *GSTA*, *GSTT*, and *GSTO* genes [57,58,59] in health outcomes associated with MSP.

The third limitation is the lack of information on the other toxic constituents of cigarette smoke other than nicotine that might lead to adverse consequences in the offspring. For example, cigarette smoke contains numerous toxic compounds, including polycyclic aromatic hydrocarbons (PAHs) and benzoquinone [60]. This group of compounds has been known to mediate several toxic effects of exposure to cigarette smoke, including childhood asthma and impaired lung function [61]. Fourth, we did not have data on the placenta samples and the expression of *GST* genes. The placenta plays a crucial role in protecting the developing fetus from harmful substances by mitigating oxidative stress [62]. GST enzymes produced by the placenta catalyze the transfer of reduced glutathione (GSH) to ROS and assorted reactive electrophilic intermediates, helping to neutralize their harmful effects by making them more water-soluble and more rapidly excreted [63].

Fifth, we focused on offspring genotypes, but maternal genotypes regarding *GST* genes were not available. Although offspring *GST* polymorphism did not seem to affect maternal levels of nicotine and its metabolites, *GST* enzymes in the mother might affect nicotine metabolites or other compounds [21] and modify the effect of exposure on offspring DNAm. Sixth, the analyses of the associations between nicotine metabolites and offspring DNAm only focused on DNAm measurement at birth. Future work may be necessary to investigate the long-term associations of MSP and DNAm measures at 10 and 18 years in this cohort.

## 5. Conclusions

The findings suggest that offspring genetic variations in *GST* genes modify the effect of tobacco smoke chemicals (nicotine metabolites in maternal sera) on offspring DNAm at a limited number of CpGs in a sex-specific manner. Importantly, the methylation of a specific CpG site, cg05575921 (associated with the *AHRR* gene), which has consistently been identified as an indicator of exposure to MSP, remained unaffected by *GST* polymorphisms. This further supports its value as a reliable biomarker for MSP exposure. Future studies are necessary to test the role of additional genetic polymorphisms in the association between MSP and offspring DNAm.

## Figures and Tables

**Table 1:** Blocks of linkage disequilibrium (LD) between genetic variants of *GST* genes.

Block of LD and *GST* Gene	*GST* SNP Variants
Block 1 (*GSTM4*, *GSTM2*)	rs506008, rs638820
Block 2 (*GSTM2*)	rs574344, rs12024479
Block 3 (*GSTM5*)	rs12736389, rs929166
Block 4 (*GSTM5*)	rs3768490, rs11807
Block 5 (*GSTM3*)	rs1537234, rs1537236, rs7483, rs10735234
*GSTP1* *	rs1695

* rs1695 (*GSTP1*) does not belong to any block and was analyzed additionally.

**Table 2:** Characteristics of the total Isle of Wight birth cohort and the analyzed samples.

	Males			Females		
	Total Cohort (N = 786)	Analyzed Samples (n = 251)	*p* Value	Total Cohort (N = 750)	Analyzed Samples (n = 242)	*p* Value
Maternal age (years)	(N = 609) 29.6 (0.32)	(n = 227) 29.5 (0.26)	0.14	(N = 584) 29.6 (0.33)	(n = 207) 29.5 (0.25)	0.12
Maternal BMI (kg/m^2^)	(N = 226) 23.7 (3.7)	(n = 198) 23.7 (3.6)	0.93	(N = 242) 24.6 (4.2)	(n = 200) 23.8(5.3)	0.69
Socioeconomic status: High Medium Low	(N = 684) 61 (8.9%) 517 (75.6%) 106 (15.5%)	(n = 247) 21 (8.5%) 187 (75.7%) 39 (15.8%)	0.06	(N = 673) 50 (7.4%) 520 (77.3%) 103 (15.3%)	(n = 240) 22 (9.2%) 189 (78.8%) 29 (12%)	0.26
MSP (yes vs. no)	(N = 778) 25.2%	(n = 251) 19.5%	0.06	(N = 743) 25.3%	(n = 242) 19.4%	0.06
Serum nicotine (nM)	(N = 288) 0.982 (2.403)	(n = 251) 0.931 (2.440)	0.71	(N = 295) 0.904 (2.251)	(n = 242) 0.842 (1.813)	0.27
Serum cotinine (nM)	(N = 288) 5.135 (8.836)	(n = 251) 4.994 (9.148)	0.71	(N = 295) 5.041 (9.456)	(n = 242) 4.354 (8.356)	0.40
Serum norcotinine (nM)	(N = 288) 0.038 (0.109)	(n = 251) 0.038 (0.105)	0.90	(N = 288) 0.043 (0.116)	(n = 242) 0.036 (0.111)	0.48
Serum hydroxycotinine (nM)	(N = 288) 0.190 (0.266)	(n = 251) 0.190 (0.234)	0.95	(N = 288) 0.191 (0.450)	(n = 242) 0.179 (0.296)	0.27

Normally distributed variables (maternal age and BMI) are represented as the mean (SD). Skewed variables (nicotine and its downstream metabolites) are presented as medians (IQR). MSP: maternal smoking in pregnancy; SES: socioeconomic status.

**Table 3:** *GST* genotypes of the total Isle of Wight birth cohort and the analyzed samples.

	Males			Females		
	Total Cohort (N = 786)	Analyzed Samples (n = 251)	*p* Value	Total Cohort (N = 750)	Analyzed Samples (n = 242)	*p* Value
rs506008 AA AG GG	(N = 576) 10 (1.7%) 134 (23.3%) 432 (75%)	(n = 233) 3 (1.3%) 53 (22.7%) 177 (76%)	0.73 *	(N = 580) 14 (2.4%) 146 (25.2%) 420 (72.4%)	(n = 227) 4 (1.8%) 50 (22%) 173 (76.2%)	0.20 *
rs574344 AA AT TT	(N = 579) 2 (0.4%) 79 (13.6%) 498 (86%)	(n = 233) 1 (0.4%) 28 (12%) 204 (87.6%)	0.49 *	(N = 586) 4 (0.7%) 86 (14.7%) 496 (84.6%)	(n = 229) 4 (1.8%) 26 (11.3%) 199 (86.9%)	0.34 *
rs12736389 CC CG GG	(N = 576) 16 (2.8%) 162 (28.2%) 398 (69%)	(n = 230) 2 (0.9%) 69 (30%) 159 (69.1%)	0.97 *	(N = 576) 17 (3%) 166 (28.8%) 393 (68.2%)	(n = 228) 9 (4%) 69 (30.2%) 150 (65.8%)	0.43 *
rs3768490 AA AC CC	(N = 575) 64 (11.1%) 245 (42.6%) 266 (46.3%)	(n = 230) 26 (11.3%) 110 (47.8%) 94 (40.9%)	0.23	(N = 579) 56 (9.7%) 267 (46.1%) 256 (44.2%)	(n = 224) 19 (8.5%) 104 (46.4%) 101 (45.1%)	0.82
rs1537234 AA AC CC	(N = 575) 102 (17.7%) 275 (47.8%) 198 (34.5%)	(n = 231) 41 (17.8%) 125 (54.1%) 65 (28.1%)	0.10	(N = 574) 101 (17.6%) 275 (47.9%) 198 (34.5%)	(n = 225) 41 (18.2%) 109 (48.4%) 75 (33.4%)	0.93
rs1695 AA AG GG	(N = 573) 253 (44.2%) 238 (41.5%) 82 (14.3%)	(n = 234) 95 (40.6%) 112 (47.9%) 27 (11.5%)	0.12	(N = 581) 222 (38.2%) 271 (46.6%) 88 (15.2%)	(n = 226) 90 (39.8%) 105 (46.5%) 31 (13.7%)	0.79

*p* values calculated for Chi square tests. * Due to the rarity of the AA genotype for rs506008, individuals with the AA genotype were combined with the AG genotype. Due to the rarity of the AA genotype for rs574344, individuals with the AA genotype were combined with the AT genotype. Due to the rarity of the CC genotype for rs12736389, individuals with the CC genotype were combined with the CG genotype.

**Table 4:** Multiple linear regression with norcotinine and hydroxycotinine, *GST* SNPs, and their interaction on CpG sites whose methylation levels are associated with nicotine and its metabolites in male and female offspring.

CpG Site and Associated Gene Name	*GST* SNPs Representing Different Genes		Serum Norcotinine		*GST* SNP		Norcotinine x *GST* SNP		Serum Hydroxycotinine		*GST* SNP		Hydroxycotinine x *GST* SNP	
			Effect Size (β)	*p*	Effect Size (β)	*p*	Effect Size (β)	*p*	Effect Size (β)	*p*	Effect Size (β)	*p*	Effect Size (β)	*p*
Males														
cg18473733 *KLF2*	rs574344	AT/AA *	-	-	-	-	-	-	−0.040	0.0001	−0.116	0.08	0.08	0.003
cg25949550 *CNTNAP2*		AT/AA *	-	-	-	-	-	-	−0.048	0.01	0.279	0.05	−0.124	0.03
cg09935388 *GFI1*	rs12736389	CG/CC *	−0.099	0.02	0.345	0.09	−0.183	0.03	-	-	-	-	-	-
cg11647108 *ANXA11*	rs1695	AA	-	-	-	-	-	-	0.473	0.0001	0.436	0.06	−0.401	0.001
		AG	-	-	-	-	-	-	0.473	0.0001	0.579	0.01	−0.415	0.001
cg01952185 *TIFAB*		AA	-	-	-	-	-	-	0.192	0.0008	0.092	0.42	−0.129	0.03
		AG	-	-	-	-	-	-	0.192	0.0008	0.216	0.05	−0.165	0.006
Females														
cg12160087 *CCDC64*	rs506008	AG/AA	-	-	-	-	-	-	−0.046	0.0001	−0.090	0.05	0.056	0.007
cg18473733 *KLF2*		AG/AA	−0.031	0.03	0.130	0.05	−0.070	0.02	-	-	-	-	-	-
cg12160087 *CCDC64*	rs1537234	AA	-	-	-	-	-	-	−0.037	0.03	−0.180	0.02	0.076	0.02
		AC	-	-	-	-	-	-	−0.037	0.03	0.024	0.60	−0.009	0.64

* Due to the rarity of the AA genotype for rs574344, for analysis, individuals with this genotype were combined with those with the AT genotype (reference: TT). Due to the rarity of the CC genotype for rs12736389, individuals with this genotype were combined with those with the CG genotype (reference: GG). Due to the rarity of the AA genotype for rs506008, individuals with the AA genotype were combined with the AG genotype. For rs1695, AA and AG genotypes had sufficient numbers to be analyzed as separate groups (reference: GG).

**Table 5:** R square for different multiple linear regressions on nicotine downstream metabolites and offspring DNAm with and without *GST* SNPs and the interaction terms between nicotine metabolites and *GST* SNPs.

CpG Site and Associated Gene Name	*GST* SNPs Representing Different Genes	Serum Norcotinine				Serum Hydroxycotinine			
		without *GST* Genes R^2^	with *GST* Genes as Covariate R^2^	Interaction of *GST* and Norcotinine R^2^	Increase in R^2 #^	without *GST* Genes R^2^	with *GST* Genes as Covariate R^2^	Interaction of *GST* and Hydroxycotinine R^2^	Increase in R^2 #^
Males									
cg18473733 *(KLF2* gene)	rs574344	-	-	-	-	0.502	0.505	0.532	5.98%
cg25949550 *CNTNAP2*		-	-	-	-	0.309	0.311	0.332	7.44%
cg09935388 *(GFI1* gene)	rs12736389	0.1688	0.1689	0.196	16.11%	-	-	-	-
cg11647108 *(ANXA11* gene)	rs1695	-	-	-	-	0.182	0.191	0.254	39.56%
cg01952185 *(TIFAB* gene)		-	-	-	-	0.190	0.200	0.241	26.84%
Females									
cg12160087 *(CCDC64* gene)	rs506008	-	-	-	-	0.1611	0.1612	0.201	24.77%
cg18473733 *KLF2* gene)		0.4362	0.4633	0.483	10.73%	-	-	-	-
cg12160087 *CCDC64* gene)	rs1537234	-	-	-	-	0.167	0.169	0.213	27.54%

Due to the rarity of the AA genotype for rs574344, individuals with this genotype were combined with those with the AT genotype (reference: TT) for analysis. Due to the rarity of the CC genotype for rs12736389, individuals with this genotype were combined with those with the CG genotype (reference: GG) for analysis. For rs1695, AA and AG SNPs had sufficient frequencies to be analyzed as separate groups (reference: GG). ^#^ An increase in R^2^ indicates the percentage increase in R^2^ from the model without *GST* SNPs to the model with *GST* SNPs and interaction terms.

## Data Availability

To ensure compliance with participants’ consent and ethical approval, data access is restricted. For inquiries regarding the data presented in this study, interested parties may contact the corresponding author.

## Supplementary Materials

The following supporting information can be downloaded at: https://www.mdpi.com/article/10.3390/genes14081644/s1. Table S1: Association of offspring *GST* gene polymorphism with nicotine and downstream metabolites in maternal sera.
