# Supplementary material for: Polymorphisms in Glutathione S-Transferase (GST) Genes Modify the Effect of Exposure to Maternal Smoking Metabolites in Pregnancy and Offspring DNA Methylation
Source: Genes (Basel). 2023 Aug 18;14(8):1644. doi: 10.3390/genes14081644 (PMC10454475; doi:10.3390/genes14081644)
Supplement: Supplementary file 1 [file genes-14-01644-s001.zip › genes-2541626-supplementary.pdf]

Supplementary Table S1. Association of offspring *GST* gene polymorphism with nicotine and its downstream metabolites in maternal sera

	Serum nicotine (nM)		Serum cotinine (nM)		Serum norcotinine (nM)		Serum hydroxycotinine (nM)	
***GST* haplotype**	**Effect estimate (β)**	**P value**	**Effect estimate (β)**	**P value**	**Effect estimate (β)**	**P value**	**Effect estimate (β)**	**P value**
**Males**								
**rs506008**	0.521	0.77	-4.858	0.71	-0.080	0.44	-2.160	0.12
**AG**	ref		ref		ref		ref	
**GG**								
**rs574344**								
**AT**	0.349	0.88	-14.904	0.37	-0.129	0.326	-2.369	0.18
**TT**	ref		ref		ref		ref	
**rs12736389**								
**CG**	-0.095	0.95	18.537	0.13	0.091	0.34	0.852	0.51
**GG**	ref		ref		ref		ref	
**rs3768490**								
**AA**	-1.072	0.67	-4.583	0.80	0.042	0.77	-0.850	0.67
**AC**	1.469	0.35	9.353	0.42	0.049	0.59	0.715	0.56
**CC**	ref		ref		ref		ref	
**rs1537234**								
**AA**	0.768	0.74	-24.208	0.15	-0.121	0.36	-1.742	0.33
**AC**	0.439	0.80	-19.149	0.14	-0.137	0.18	-1.551	0.26
**CC**	ref		ref		ref		ref	
**rs1695**								
**AA**	0.544	0.83	-9.800	0.59	-0.082	0.56	-1.484	0.44
**AG**	0.405	0.87	7.663	0.67	0.066	0.64	0.394	0.83
**GG**	ref		ref		ref		ref	
**Females**								
**rs506008**								
**AG**	-1.660	0.27	-9.101	0.20	-0.059	0.48	-1.019	0.39
**GG**	ref		ref		ref		ref	
**rs574344**								
**AT**	1.206	0.51	-5.85	0.52	-0.055	0.62	-0.834	0.59
**TT**	ref		ref		ref		ref	
**rs12736389**								
**CG**	0.837	0.53	-0.737	0.91	0.002	0.98	-0.229	0.84
**GG**	ref		ref		ref		ref	
**rs3768490**								
**AA**	-3.430	0.17	-6.969	0.50	0.082	0.50	0.044	0.98
**AC**	1.022	0.44	4.483	0.42	0.029	0.65	0.049	0.97
**CC**	ref		ref		ref		ref	
**rs1537234**								
**AA**	1.083	0.59	12.027	0.23	0.182	0.13	2.888	0.09
**AC**	0.454	0.76	12.371	0.10	0.174	0.06	2.464	0.06
**CC**	ref		ref		ref		ref	
**rs1695**								
**AA**	1.1541	0.58	6.684	0.50	0.031	0.79	0.053	0.97
**AG**	1.928	0.35	6.366	0.52	0.061	0.60	0.808	0.62
**GG**	ref		ref		ref		ref	

Due to the rarity of the AA genotype for rs506008, individuals with the AA genotype were combined with the AG genotype. Due to the rarity of the AA genotype for rs574344, individuals with the AA genotype were combined with the AT genotype. Due to the rarity of the CC genotype for rs12736389, individuals with the CC genotype were combined with the CG genotype.
